# Strategies to promote the implementation of Screening, Brief Intervention, and Referral to Treatment (SBIRT) in healthcare settings: a scoping review

**DOI:** 10.1186/s13011-021-00380-z

**Published:** 2021-05-11

**Authors:** Kelli Thoele, Laura Moffat, Stephanie Konicek, Monika Lam-Chi, Erica Newkirk, Janet Fulton, Robin Newhouse

**Affiliations:** 1 Robert Wood Johnson Future of Nursing Scholar, Indiana University School of Nursing, 600 Barnhill Drive, Indianapolis, IN 46202 USA; 2 Sigma Theta Tau International Rising Star of Research and Scholarship, Indiana University Health Arnett Hospital, Purdue University, 5165 McCarty Lane, Lafayette, IN 47905 USA; 3 Indiana University Health, South Central Region, 601 West 2nd Street, Bloomington, IN 47403 USA; 4 Indiana University Health, Fairbanks Hall, 340 West 10th Street, Suite 3200, Indianapolis, IN 46202 USA; 5 Indiana University Health West, 1111 Ronald Reagan Parkway, Avon, IN 46123 USA; 6 Indiana University School of Nursing, 600 Barnhill Drive, NU 132, Indianapolis, IN 46202 USA; 7 Indiana University School of Nursing, Distinguished Professor, Indiana University, 600 Barnhill Drive, NU 132, Indianapolis, IN 46202 USA

**Keywords:** Implementation, Implementation strategies, Screening, brief intervention, referral to treatment (SBIRT), Substance-related disorders, Scoping review

## Abstract

**Background:**

Screening, brief intervention, and referral to treatment (SBIRT), is an approach for the prevention and treatment of substance use disorders, but is often underutilized in healthcare settings. Although the implementation of SBIRT is challenging, the use of multi-faceted and higher intensity strategies are more likely to result in the successful incorporation of SBIRT into practice in primary care settings. SBIRT may be used in different healthcare settings, and the context for implementation and types of strategies used to support implementation may vary by setting. The purpose of this scoping review is to provide an overview regarding the use of strategies to support implementation of SBIRT in all healthcare settings and describe the associated outcomes.

**Methods:**

A scoping review was conducted using CINAHL Complete, HealthBusiness FullTEXT, PsycINFO, PubMed, and Embase to search for articles published in English prior to September 2019. The search returned 462 citations, with 18 articles included in the review. Two independent reviewers extracted data from each article regarding the theory, design, timeline, location, setting, patient population, substance type, provider, sample size and type, implementation strategies, and implementation outcomes. The reviewers entered all extracted data entered into a table and then summarized the results.

**Results:**

Most of the studies were conducted in the United States in primary care or emergency department settings, and the majority of studies focused on SBIRT to address alcohol use in adults. The most commonly used strategies to support implementation included training and educating stakeholders or developing stakeholder interrelationships. In contrast, only a few studies engaged patients or consumers in the implementation process. Efforts to support implementation often resulted in an increase in screening, but the evidence regarding the brief intervention is less clear, and most studies did not assess the reach or adoption of the referral to treatment.

**Discussion:**

In addition to summarizing the strategies used to increase reach and adoption of SBIRT in healthcare settings, this scoping review identified multiple gaps in the literature. Two major gaps include implementation of SBIRT in acute care settings and the application of implementation theories to inform healthcare efforts to enable use of SBIRT.

## Background

More than 20 million people aged 12 and older in the United States have a substance use disorder [1]. Substance use disorders (SUD), defined as health problems, disability, and failure to meet responsibilities caused by alcohol or drug use [1], have a significant impact on individuals, families, and communities. In addition to healthcare costs associated with the treatment of comorbidities, a projected $42 billion will be spent on SUD treatment in 2020 [2]. When including direct and indirect costs related to crime and lost worker productivity, the national cost of substance abuse increases to $740 billion annually [3]. Despite the known consequences of SUD, healthcare providers rarely use validated tools to screen patients for SUD, and only 11% of people who need substance use treatment receive treatment at a specialty facility [1, 4].

Screening, Brief Intervention, and Referral to Treatment (SBIRT) is a comprehensive public health approach to delivering care for individuals who have or are at risk of developing SUD [5]. SBIRT is a three-step process that involves 1) using a validated tool to screen patients to assess the severity of substance use, 2) providing a brief intervention when indicated by screening and clinical judgment, and 3) providing a referral to treatment when appropriate [5]. The receipt of SBIRT is associated with reductions in alcohol and illicit drug use [6] and diminished societal costs related to automobile accidents, arrests, incarcerations, work absences, and other factors [7, 8]. Compared to usual care, a brief intervention is effective in the reduction of alcohol consumption [9], although the evidence for other substances is unclear [9, 10]. Several organizations recognize the potential of SBIRT in addressing substance use [11–14]. Despite the potential benefits of using SBIRT in clinical practice, this intervention is underutilized in healthcare settings representing a substantial gap in implementation. Clinicians report that they infrequently use screening tools to screen for substance use, and clinical students may not have preceptors with SBIRT experience [4, 15]. Additionally, less than 2% of pediatric emergency physicians report consistent use of SBIRT for adolescents with alcohol-related emergency department visits [16].

When an intervention is underutilized in clinical practice, the next step is to study the implementation of that intervention [17, 18]. Greenhalgh et al. define implementation as “active and planned efforts to mainstream an innovation within an organization.”^19,p. 582^ This process includes the decision to use an intervention (described using the terms adoption, assimilation, acceptance, and uptake) [19–22], and continued use of the intervention (described using the terms sustainment and maintenance) [20, 21, 23]. The methods used to enhance adoption, implementation, and sustainment of a new practice are referred to as implementation strategies [24]. Implementation strategies may include activities such as training and educating stakeholders, adapting the intervention to fit the context, or providing interactive assistance during the implementation process [25, 26]. While there are several different measures to determine the outcomes associated with implementation [27], the outcomes in this review include *reach* (i.e., the proportion of patients who received the intervention) and *adoption* (i.e., the proportion of individual providers, groups, or organizations that decided to use the intervention) [28, 29].

Previous reviews on the use of strategies to support the implementation of SBIRT have focused on unhealthy alcohol use within primary care settings and were published in 2005 and 2016. These analyses indicated that the use of multi-faceted strategies that addressed a combination of patients, professionals, and organizations, was more effective than the use of strategies that only addressed the healthcare professionals [30]. These studies additionally found that a higher intensity of an implementation strategy (e.g., amount of training) was associated with greater efficacy of implementation of a brief alcohol intervention in primary care [31].

Prior reviews are limited to the primary care setting, but SBIRT can be used in other settings such as acute care and emergency departments which may have different contexts for implementation than primary care. Investigators have studied the implementation of SBIRT in various healthcare settings, and an understanding of the strategies used to increase the number of patients who receive SBIRT and providers who use SBIRT in various contexts may inform future research and clinical practice. Therefore, the research question guiding this research is, “What implementation strategies are used to increase the reach and adoption of SBIRT when implementing SBIRT in healthcare settings, and what are the associated outcomes related to reach and adoption?” Scoping reviews are used to map the current field of study and identify gaps in the existing literature [32, 33]. Scoping reviews include a systematic search and summary of the existing literature; however, unlike a systematic review, the scoping review method does not include an assessment of the risk of bias within each study or synthesis of the evidence [33]. A scoping review was therefore determined most appropriate, as this method will provide an overview of the evidence. The purpose of this scoping review is to provide an overview of existing evidence regarding the use of implementation strategies to promote the implementation of SBIRT in healthcare settings.

## Methods

Investigators used a scoping review method as described by Arksey and O’Malley [32]. This method includes identifying a research question, identifying and selecting studies, extracting data, and then collating and summarizing results [32].

### Identifying a research question

The investigators noted a gap in the literature and established the research question. The investigators developed but did not publish a protocol to conduct the review and answer this research question.

### Identifying and selecting studies

To be included in this review, articles had to be published in English, contain empirical evidence, address the implementation of SBIRT in healthcare settings, describe strategies to promote implementation, and measure an outcome of interest (i.e., reach or adoption of SBIRT). Additionally, there had to be a comparison of the outcome, such as pre-intervention and post-intervention data, longitudinal data, or comparison to a control group. Exclusion criteria included abstracts, posters, dissertations, or articles that used SBIRT for something other than unhealthy substance use. These inclusion and exclusion criteria were selected to obtain evidence to address the purpose of the review and to summarize evidence regarding the changes in reach and adoption related to the use of implementation strategies.

The articles for this review were identified through a literature search, using the key terms “SBIRT” OR “screening brief intervention referral to treatment” AND multiple terms related to implementation (adopt*, assimilation, acceptance, uptake, implement*, sustain*, maintenance). Because not all authors use the term ‘strategy’ when describing methods to enhance implementation, this term was not included in the search. Databases for the search included CINAHL Complete, HealthBusiness FullTEXT, PsycINFO, PubMed, and Embase. These databases were selected to capture nursing, healthcare administration, behavioral science literature, and international literature. Publication dates were not limited, and the literature search was conducted on August 31, 2019. A health science librarian provided feedback on the search strategy prior to the completion of the literature search.

The initial screening process included a review of all titles and abstracts and then removal of the citations that clearly did not meet the criteria for inclusion in the review. After obtaining the full text for all of the remaining citations, the investigator then removed all non-English articles, abstracts, posters, and dissertations. The remaining full-text articles were then screened for inclusion in the review using a screening tool that listed the inclusion and exclusion criteria.

### Extracting data

Variables of interest for this review included the study theory or framework, design and timeline, location and setting, patient population, substance type, the type of providers using SBIRT, sample size and type, implementation strategies used, and implementation outcomes. Most of the variables (theory/framework, design and timeline, location, setting, population, substance type, and providers using SBIRT) were extracted directly from the articles. When the study authors did not clearly state the study design, the reviewers selected a design to describe the study. The sample size and type were extracted directly from the article, with a focus on the sample size included in the final data analysis. When the study authors did not provide the exact sample size, the reviewers described the sample size based on information in the article.

To identify implementations strategies, the reviewers looked for descriptions of methods to facilitate adoption, implementation, or sustainment of SBIRT, such as training, adapting the intervention, providing ongoing support, or providing financial incentives. The implementation strategies described in each article were extracted and then categorized by the reviewers into categories, as defined and described by Powell et al. [25] and Waltz et al. [26] When reviewing the articles, research activities, such as data collection for research purposes and data analysis, were not considered to be implementation strategies. Funding and academic/practice partnerships were included as implementation strategies when they were explicitly mentioned in the article but were not included based on the acknowledgments section or authors’ credentials or places of employment.

The outcomes of reach and adoption were extracted from each article. Although adoption is generally defined as a cognitive decision [22], researchers often measure self-reported behavior or actual behavior as a proxy for the adoption decision. For this review, reviewers extracted adoption data on providers’ intention to use SBIRT or behavior regarding SBIRT. Study outcomes other than reach or adoption (e.g., provider attitude, knowledge, patient use of substances after receiving SBIRT) were not extracted from the articles. When extracting outcomes related to the brief intervention, reviewers also included different terms used to describe a brief intervention, such as ‘brief advice,’ ‘motivational interviewing,’ and ‘counseling.’

A data collection instrument was developed and built into Qualtrics XM®, a cloud-based survey software tool, with pilot testing completed prior to use. This tool was used to guide data extraction, collect and organize data from each article, and compare reviewer responses. Once reviewers determined that an article met criteria for inclusion in the review, each article was independently reviewed by the primary investigator and a second reviewer. Both reviewers entered data into the Qualtrics tool. The study timeline was not included in the original data collection tool, and this variable was extracted later in the scoping review process. At the completion of the independent reviews, all discrepancies were discussed by the two independent reviewers. All unresolved discrepancies were then brought to one of two additional investigators, who then made a final determination. One study author was contacted to clarify the substance type addressed in an article. In alignment with the scoping review methods described by Arksey & O’Malley [32], reviewers did not appraise the quality of each article.

### Collating and summarizing results

Once consensus was reached, the results were entered into a table in Microsoft Word to collate the results and summarize the data. The reviewers met in person to summarize the information, and all investigators provided additional input via email or in-person discussions.

## Results

**Fig. 1:**
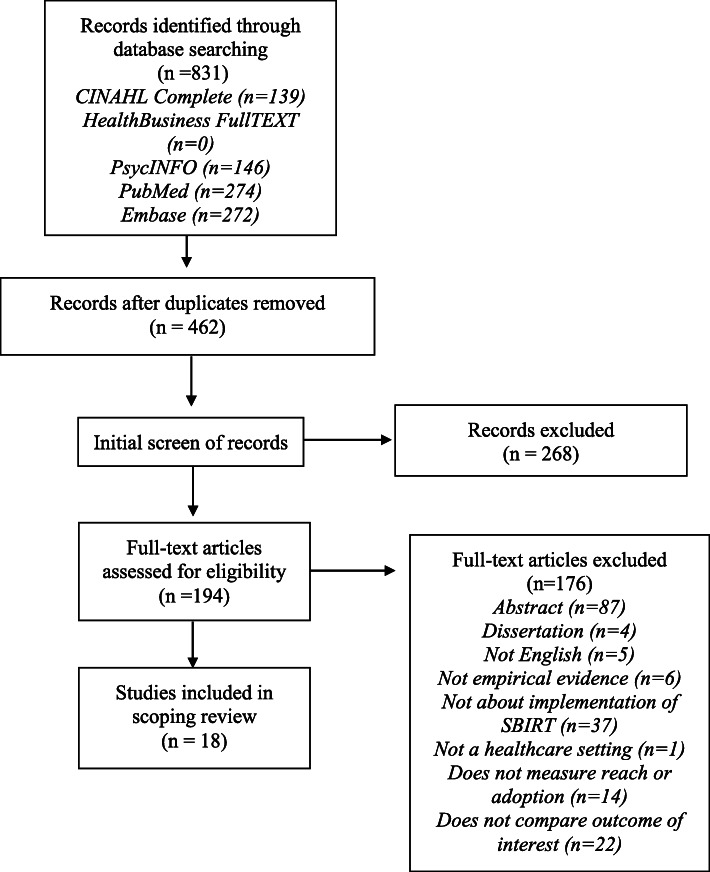
Flow Diagram of Study Selection

The literature search identified 462 unique records after the removal of duplicates. Two hundred sixty-eight articles were excluded based on a review of the titles and abstracts, and then a review of full-text citations led to the exclusion of abstracts, dissertations, and non-English articles. Two reviewers assessed the remaining 102 full-text articles for eligibility based on previously noted inclusion and exclusion criteria. The search concluded with 18 articles identified for in-depth review (see Fig. 1).

### Study characteristics

The majority of studies (*n* = 15) did not state a specific theory or framework; however, investigators of the remaining three studies noted the use of the following frameworks: Framework for Design and Evaluation of Complex Interventions to Improve Health, the Consolidated Framework of Implementation Research, and Knowledge Translation. The most common study designs were pre-post studies, randomized controlled trials, longitudinal studies, and quality improvement, with the timeframe of the studies ranging from 30 days to 5.5 years. Thirteen studies were conducted in the United States, followed by Europe (*n* = 3), Canada (*n* = 1), and Australia (n = 1). The most common settings were primary care and emergency departments/trauma centers. The majority of the included patient populations were adults and/or trauma patients (*n* = 10), although three of the studies addressed the implementation of SBIRT in the adolescents and pediatric populations. More than half of the studies were implementing SBIRT to address alcohol use, while the remaining studies focused on SBIRT to address alcohol and other drugs, tobacco, or all substance types. SBIRT was generally provided by multiple professions within each study, although the studies in which only one profession provided SBIRT generally focused on physicians (see Table 1).

**Table 1:** Key Features of Included Studies

Article	Framework	Design and Timeline	Location	Setting	Population	Substance Type	Who is providing SBIRT	Sample	Outcomes
Anderson et al., 2016 [43]	None	Cluster randomized 2x2x2 factorial trial *4 weeks (baseline)* *12 weeks (implementation)*	Catalonia, England, the Netherlands, Poland, Sweden	120 primary healthcare centers	Adults	Alcohol	Providers (general practitioners, nurses, or other professionals)	Approximately 5000–20,000 registered patients at the healthcare centers Average of 1500 consultations at each center per month	Screening significantly increased in groups that received training/support (Groups 2, 5, 6, 8) compared to groups that did not. Screening significantly increased in groups who received financial reimbursement (Groups 3,5,7, 8) compared to groups that did not. Not a significant increase in screening for the groups that received the electronic brief intervention (Groups 4, 6, 7, 8) compared to groups that did not.
Bendsten et al., 2016 [45]	None	Subanalysis of a randomized controlled trial (Anderson et al., 2016) *4 weeks (baseline)* *12 weeks (implementation)*	Catalonia, England, the Netherlands, Poland, Sweden	120 primary healthcare centers	Adults	Alcohol	Providers (general practitioners, nurses, or other professionals)	Approximately 5000–20,000 registered patients at the healthcare centers Average of 1500 consultations at each center per month	Not a significant increase in screening for the groups that received the electronic brief intervention (Groups 4, 6, 7, 8) compared to groups that did not. Significant increase in proportion of patients who received brief advice in the sample as a whole (70 to 80%, *p* < 0.05).
Bernstein et al., 2007 [51]	None	Pre-post- repeated measures design *12 months*	United States	14 academic emergency departments	Emergency department patients	Alcohol	Providers (physicians, registered nurses, advanced practice providers, social workers, and other staff)	288 providers	Significantly higher utilization of SBIRT skills 3 months (*p* < 0.001) and 12 months (*p* < 0.001) after receiving education, when compared to baseline. Providers reported higher utilization of SBIRT skills at 3 months than 12 months.
Egizio et al., 2019 [50]	None	Pre-post^a^ *30 days*	United States	Field placement of supervisors (e.g., family service agencies, hospitals, community clinics, housing programs)	All patients coming in contact with field supervisors	Alcohol and other drugs	Social workers who provided field supervision to social work students delivering SBIRT	74 field supervisors	Increase in the percentage of supervisors who used motivational interviewing (73.9 to 86.5%) and SBIRT (17.4 to 43.2%) when comparing baseline to 30 days after training.
Henihan et al., 2016 [44]	Framework for Design and Evaluation of Complex Interventions to Improve Health	Randomized controlled pre-and-post design *3 months*	Ireland	15 primary care facilities	Adults receiving addiction treatment with an opioid agonist	Alcohol	General practitioners	81 patients (34 in the intervention group and 47 in the control group)	A higher percentage of patients in the intervention group were screened (53% versus 26%), received a brief intervention (47% versus 19%) and received a referral to treatment (3% versus 0%) when compared to the control group.
Lapham et al., 2012 [49]	None	Retrospective, natural history study *12 months (baseline)* *3 months (transition)* *3 months (implementation)* *9 months (dissemination)*	United States	Outpatient Veteran Affairs facilities	Veterans	Alcohol	Providers	6788 patients who screened positive for alcohol misuse	The percentage of patients receiving a brief intervention increased significantly over time from 5.5 to 29% (p < 0.001).
Lindholm et al., 2010 [34]	None	Pre-post^a^ *12 months (pre-intervention)* *12 months (post-intervention)*	United States	18 primary care clinics	Adults	Tobacco	Medical assistant completed screening, clinicians provided brief intervention	502,359 patients (255,138 pre-intervention and 247,221 post-intervention)	Statistically significant increase in documentation of smoking status from 71.6 to 78.4% (p < 0.001). Pre-intervention data not available for brief intervention or referral to treatmen.t
Mello et al., 2009 [42]	None	Quality improvement^a^ *1 month* *(baseline)* *6 months* *(Phase 1)* *6 months* *(Phase 2)*	United States	1 community hospital emergency department	Not a specific population	Alcohol	Physicians, physician assistants, and nurse practitioners provided the screening and referral. Research assistants provided the brief intervention	1509 patients (254 baseline, 922 when research assistant was in the emergency department during the study, 333 patients one month after the research assistant was no longer present)	Screening by emergency department staff increased from 50% (baseline) to 71% (when research assistant was present), then back to 50% after research assistant was no longer present.
Mello et al., 2013 [35]	None	Longitudinal^a^ *12 months (adoption)* *12 months (implementation)* *12 months (maintenance)*	United States	7 pediatric trauma centers	Admitted adolescent trauma patients	Alcohol	Differed at each site, but in general, nurses completed screening and social workers provided brief intervention and decided on referral to treatment	400 patients (160 baseline, 116 in implementation phase, 124 in maintenance phase)	The percentage of patients screened increased from 11% (baseline) to 73% (implementation and maintenance phases).
Mertens et al., 2015 [46]	None	Cluster randomized implementation trial *12 months*	United States	54 primary care clinics	Adults	Alcohol	Arm 1: Physicians Arm 2: Non-physician providers (i.e., clinical health educators, behavioral medicine specialists, nurses) and medical assistants Arm 3: Usual care	Average number of visits per month= 35,519 patients in Arm 1, 34,167 patients in Arm 2, 31,935 patients in Arm 3	Screening was highest in Arm 2 (51%) compared to Arm 1 (9%) and Arm 3 (3.5%). For patients screening positive, the brief intervention and referral was highest in Arm 1 (44%) compared to Arm 2 (3.4%) and Arm 3 (2.7%).
Muench et al., 2015 [36]	None	Longitudinal^a^ *2 years*	United States	6 primary care clinics	Adults	Alcohol and other drugs	Receptionists gave annual screen to patients at check-in Medical assistants scored the screen, and if indicated, completed a more detailed brief assessment Clinicians (physician, physician’s assistant, nurse practitioner) performed the brief intervention	Approximately 11,000 patients each quarter	Screening rates significantly increased over time, with a median increase of 6.4% between quarters (p < 0.05). Brief assessment rates (AUDIT and/or DAST) increased over time, with a median increase of 7.0% between quarters (*p* < 0.05). Brief intervention rates decreased over time, with a decrease of 3.7% between quarters. A non-significant trend (*p* > 0.05).
Rieckmann et al., 2018 [37]	Consolidated Framework of Implementation Research	Longitudinal mixed- methods design *30 months (pre-implementation)* *6 months (transition period)* *30 months (post-implementation)*	United States	Primary care	18–64 year old Medicaid recipients enrolled in a coordinated care organization	Alcohol and other drugs	Unknown	516,708 members in the study population	Quantitative analysis revealed a significant increase in SBI rates from 0.1% of patients (baseline) to 4.6% of patients (last six months of study). Qualitative analysis revealed the importance of aligning incentives, workflow redesign, and leadership facilitation.
Salvalaggio et al., 2015 [47]	Knowledge Translation	Non-randomized, pre-post, quasi-experimental intervention design *6 months (patient-level implementation)* *6 months (provider access to knowledge translation resources)*	Canada	3 primary care networks, 3 emergency departments, 3 residency programs	Patients who received care in socio-economically disadvantaged neighborhoods	Alcohol and other drugs	Physicians/residents	64 physicians/residents (39 in the intervention group and 25 in the control group)	Overall, physicians reported that they were more likely to screen (*p* = 0.008) and refer for treatment (*p* = 0.017) after 12 months. Exposure to the intervention predicted brief intervention behavior (p < 0.05) but not screening or referral behavior.
Sharifi et al., 2014 [41]	None	Pre-post study *3 months (pre-intervention)* *1 month (intervention)* *3 months (post-intervention)*	United States	1 pediatric primary care clinic	Parents (of pediatric patients ≤12 years old) who smoke	Tobacco	Physicians/residents	3919 patients (2024 pre-intervention and 1895 post-intervention)	Not a significant change in screening. There was a significant increase in counseling for parents who screened positive.
Sterling et al., 2015 [48]	None	Cluster randomized controlled trial *2 years*	United States	1 pediatric primary care system	Adolescents 12–18 years old	Alcohol, tobacco, other drugs	Arm 1: Pediatricians Arm 2: Pediatricians and embedded behavioral health care practitioners Arm 3: Usual care	1871 patients (584 in Arm 1, 671 in Arm 2, 616 in Arm 3)	In Arm 1, pediatricians who attended 2+ trainings assessed more patients than pediatricians who attended fewer trainings(p < 0.001) and provided more brief interventions (p < 0.001) than pediatricians who attended fewer trainings. The total number of assessments in Arm 1 and Arm 2 were not significantly different. Arm 1 and Arm 2 provided significantly more brief interventions than Arm 3 (*p* < .001) Arm 1 provided more brief interventions related to substance use than Arm 2 (*p* < 0.001). Arm 2 had significantly lower referral to treatment when compared to usual care (*p* = 0.006), but Arm 1 was not significantly different from usual care
Thomas et al., 2016 [38]	None	Quality improvement (using Plan-Do-Study-Act) *12 months*	United States	1 emergency department and hospital	Adult trauma patients	Alcohol and other drugs	Multiple roles provided SBIRT (including nurses and health education specialists), and the process changed throughout the project	1664 patients	The percentage of patients who were screened significantly increased over time from 47% (Quarter 1) to 86.1% (Quarter 2) (p < 0.001) Specialist-delivered SBIRT (assessment and brief intervention when applicable) did not significantly change over time.
Whitty et al., 2015 [39]	None	Mixed-method, uncontrolled, pre-post trial *6 months (pre-intervention)* *13 months (implementation)* *6 months (post-intervention)*	Australia	1 hospital	Patients treated for alcohol-related injury and maxillofacial trauma; the majority of patients who met criteria at this hospital were Indigenous	Alcohol	Not specified (the best practice pathway was designed for medical, surgical and nursing departments)	144 patients (76 pre and 68 post)	Screening significantly increased from 9 to 81% of patients (p ≤ 0.001). No significant change in brief intervention, internal referral, or external referral.
Zimmermann et al., 2018 [40]	None	Quality improvement^a^ *8 months*	United States	1 trauma center	Trauma patients 15+ years old	Alcohol	Blood alcohol levels used as a screening tool; if a patient screened positive (blood alcohol level > 0.02%) the social worker provided a brief intervention and evaluated for treatment services	693 patients	Screening increased from 30% (month 1) to 100% (months 4–8).

^a^ = Authors did not state the design

### Implementation strategies

The authors of each study described the use of multiple strategies to support the implementation of SBIRT. Nearly every study used strategies to train and educate stakeholders (*n* = 17). Training and education included the development and distribution of educational materials, as well as the provision of in-person training ranging from 5 min to 1 full day. While training and education were used most often, the next most common approach was the development of stakeholder interrelationships (*n* = 12). Studies described developing these relationships through the identification of champions, development of interdisciplinary teams, and collaboration with researchers and other stakeholders (see Table 2).

**Table 2:** Implementation Strategies and Categories

		Use evaluative and iterative strategies	Provide interactive assistance	Adapt and tailor to context	Develop stakeholder interrelationships	Train and educate stakeholders	Support clinicians	Engage consumers	Utilize Financial Strategies	Change infrastructure
**Article**	**Implementation Strategies**	**Implementation Strategy Categories**								
Anderson et al., 2016 [43]	Conducted one (10–30 min) telephone support call (Groups 2, 5, 6, 8)		x							
	Offered an option to refer patients to an online brief intervention as an alternative to face-to-face intervention (Groups 4, 6, 7, 8)			x						
	Distributed educational materials (Groups 1, 2, 4, 5, 6, 7, 8) Asked providers to screen patients (Groups 1, 2, 4, 5, 6, 7, 8) Provided two (1–2 h) in-person trainings (Groups 2, 5, 6, 8)					x				
	Provided financial reimbursement for screening and advice activities (Groups 3, 5, 7, 8)								x	
	Provided a record sheet to document SBIRT (Groups 1, 2, 4, 5, 6, 7, 8)									x
Bendsten et al., 2016 [45]	Conducted one (10–30 min) telephone support call (Groups 2, 5, 6, 8)		x							
	Offered an option to refer patients to an online brief intervention as an alternative to face-to-face intervention (Groups 4, 6, 7, 8)			x						
	Distributed educational materials (Groups 1, 2, 4, 5, 6, 7, 8) Asked providers to screen patients (Groups 1, 2, 4, 5, 6, 7, 8) Provided two (1–2 h) in-person trainings (Groups 2, 5, 6, 8)					x				
	Provided financial reimbursement for screening and advice activities (Groups 3, 5, 7, 8)								x	
	Provided a record sheet to document SBIRT (Groups 1, 2, 4, 5, 6, 7, 8)									x
Bernstein et al., 2007 [51]	Provided technical assistance Facilitated learning of individual clinicians		x							
	Tailored brief intervention and referral resources to meet local needs			x						
	Partnered with research team and other stakeholders at each site				x					
	Provided one (2-h) interactive workshop or a web-based learning module Developed and distributed educational materials					x				
	Collaborated with volunteers from Alcoholics Anonymous							x		
Egizio et al., 2019 [50]	Provided monthly implementation support Facilitated clinical supervision		x							
	Tailored plan to address limited training and clinical supervision for SBIRT			x						
	Identified champions (i.e., field supervisors) and partnered with instructors				x					
	Provided one (1-day) training for field supervisors					x				
	Received grant to develop SBIRT certificate program								x	
Henihan et al., 2016 [44]	Partnered with the research assistant who conducted practice visits				x					
	Distributed training and educational materials Demonstrated intervention implementation Provided educational support after the workshop					x				
Lapham et al., 2012 [49]	Monitored quarterly facility-level reports	x								
	Disseminated clinical reminders via the electronic medical records						x			
	Linked performance measure to financial incentives for clinical leaders								x	
	Created a national performance measure for a brief intervention for patients with alcohol misuse									x
Lindholm et al., 2010 [34]	Completed pilot tests before wide-scale implementation	x								
	Developed a team of representatives from healthcare system (including a physician champion) and a university-based tobacco dependence research center				x					
	Provided one (20-min) onsite training and an additional visit if needed					x				
	Listed interventions in the electronic medical record if patient indicated an interest in quitting						x			
	Modified the electronic medical record to improve identification and treatment of tobacco use									x
Mello et al., 2009 [42]	Adapted plan to community emergency department environment (Phase 1) Continued ongoing exploration and adaptation (Phases 1, 2)			x						
	Met with stakeholders to obtain feedback on intervention and implementation plan (Phase 1)				x					
	Provided one (5-min) initial training of staff (Phase 2) Provided small laminated reference cards (Phase 2)					x				
	Partnered with the research assistant, who provided the brief intervention (Phase 2)						x			
Mello et al., 2013 [35]	Assessed for readiness and created an SBIRT policy (Adoption phase)	x								
	Facilitated monthly conference calls (Adoption and implementation phases) Hosted a web site for technical assistance (Adoption and implementation phases)		x							
	Identified and prepared site leaders (Adoption phase)				x					
	Provided online curriculum and in-person workshop (Adoption phase) Provided another in-person workshop and webinar on the brief intervention (Implementation phase)					x				
Mertens et al., 2015 [46]	Reviewed quality feedback reports and addressed challenges (Arms 1 and 2) Emailed quarterly reports of SBIRT rates to each clinic (Arms 1 and 2)	x								
	Provided in-person technical assistance and facilitation (Arms 1 and 2)		x							
	Provided one (2-h) initial training and one (30-min) booster training (Arms 1 and 2) Posted educational videos on intranet site (Arms 1 and 2) Provided one (1-h) training for medical assistants (Arm 2) Provided an on-demand 30-min webinar session (Arm 3)					x				
	Obtained public support from leaders (Arms 1 and 2) Directed the medical assistants to use the tool (Arm 2) Added screening questions to the electronic health record to facilitate SBIRT (Arms 1, 2, 3)									x
Muench et al., 2015 [36]	Adapted the process to the workflow at each site			x						
	Designated champions at each site				x					
	Provided one (3.5-h) training for residents and shorter training for faculty physicians and clinic staff					x				
	Created reminders in the electronic health record to alert clinicians						x			
	Received funding from the Substance Abuse and Mental Health Services Administration								x	
	Created documentation flow sheets in the electronic health record									x
Rieckmann et al., 2018 [37]	Identified champions				x					
	Developed the workforce					x				
	Selected screening and brief intervention as incentive metrics Aligned incentives								x	
	Redesigned workflow									x
Salvalaggio et al., 2015 [47]	Completed a baseline needs assessment	x								
	Used a web platform to centralize materials Provided implementation support		x							
	Toured other sites Identified champions				x					
	Provided one (2–3 h) workshop Distributed educational materials Provided online modules and links to resources					x				
	Provided point-of-care tools to remind clinicians of SBIRT and available resources						x			
	Partnered with community members with lived experiences, who discussed scenarios and answered questions during workshops							x		
Sharifi et al., 2014 [41]	Completed a baseline needs assessment	x								
	Provided one (15-min) training session					x				
	Embedded a reminder and decision support tool in the electronic medical record Simplified the education and referral process						x			
Sterling et al., 2015 [48]	Provided feedback on rates of screening and referral each quarter and reviewed protocol and skills to promote use of SBIRT (Arms 1, 2)	x								
	Provided technical assistance and clinical consultation (Arms 1, 2)		x							
	Provided three (60-min) training sessions (Arm 1) Provided one (60-min) training session (Arm 2)					x				
	Shifted tasks of brief intervention and referral to treatment to the behavioral health care practitioner when indicated (Arm 2) Informed pediatricians of tools in the electronic medical records (Arms 1, 2, 3) Reminded pediatricians to document clinical activities (Arms 1, 2, 3)						x			
Thomas et al., 2016 [38]	Presented data monthly	x								
	Tailored implementation strategies based on identified barriers			x						
	Assembled an interdisciplinary SBIRT committee that met monthly Identified an SBIRT champion				x					
	Provided brief in-service training meetings					x				
	Designated SBIRT health education specialist to screen all patients and contact trauma resident daily						x			
	Received funding from the Substance Abuse and Mental Health Services Administration								x	
	Integrated an order for an SBIRT consult into the trauma order set									x
Whitty et al., 2015 [39]	Adapted the implementation approach and training materials to the local setting and Indigenous population			x						
	Developed resources in collaboration with consultants and other experts				x					
	Developed best practice pathway and other resources Provided six (1-h) workshops					x				
	Collaborated with an Indigenous reference group to develop the resources							x		
Zimmermann et al., 2018 [40]	Reported status updates at monthly meetings	x								
	Assembled a multidisciplinary team and developed a process for SBIRT				x					
	Provided one (4-h) training for social workers Provided an in-service to all key staff					x				
	Disseminated a list of eligible patients daily and kept this list in a project binder						x			

Half of the studies described strategies to support clinicians (*n* = 9), such as embedding reminders into the electronic health record and shifting tasks from one role (e.g., physician) to a different role (e.g., research assistant, health education specialist, or behavioral health care practitioner). Other strategies used included the use of evaluative and iterative strategies to support implementation (n = 9), such as the use of monthly or quarterly reports to summarize data, and the completion of a baseline needs assessment to assess for readiness for the implementation of SBIRT (see Table 2).

The remaining categories of implementation strategies were used in fewer than half of the studies. These included the use of interactive assistance to support implementation (*n* = 8) by providing technical assistance, conducting one-time or monthly conference calls, or by providing ongoing support, facilitation, and supervision. Several studies also described adapting and tailoring the intervention or implementation plan to the local context (n = 8). Implementation leaders most commonly tailored the resources, intervention, process, and training materials to meet the local needs or to fit into a specific setting (e.g., community emergency department) or specific population (e.g., Indigenous people). Another approach included the use of strategies to change infrastructure (n = 8). The most common infrastructure change was the modification of the electronic health record to incorporate SBIRT into the documentation. Several studies described the use of financial strategies (*n* = 7) to increase the use of SBIRT. Financial strategies included receiving funding to support the implementation of SBIRT or providing incentives or reimbursement for the use of SBIRT. Finally, a few studies described the engagement of consumers to support implementation (*n* = 3) by partnering with people with unhealthy substance use or people from a specific population (i.e., Indigenous people) to develop resources and train providers (see Table 2).

### Implementation outcomes

The majority of the studies in this review measured the percentage of patients who received the intervention (*n* = 15), while one of these studies additionally measured differences in adoption among providers. The remaining three studies measured the self-reported use of SBIRT by providers. Most of the studies in this scoping review evaluated outcomes related to screening (n = 15), followed by brief intervention (*n* = 10), referral to treatment (*n* = 4), brief intervention/referral to treatment (n = 1), and SBIRT overall (*n* = 2) (see Table 1).

#### Screening

##### Reach

Of the 15 studies measuring outcomes related to screening patients with a valid and reliable tool, most of the studies measured *reach*, or the percentage of patients who received screening (*n* = 13). Most of these studies (*n* = 9) utilized the same implementation strategies for all study participants via a quality improvement, pre-post, or longitudinal study design. In these studies, screening generally increased over time [34–40], but three studies did not report if this increase was significant [35, 37, 40]. Only one study, which focused on parents of patients rather than patients, reported no change in screening [41]. Another study reported an increase in screening when a research assistant was present, then a return to baseline when the research assistant was no longer present [42].

The remaining studies (*n* = 4) divided participants into groups and evaluated outcomes using randomized controlled, randomized controlled pre-post, or non-randomized pre-post quasi-experimental designs. The use of training [43, 44] and financial reimbursements [43] resulted in significant increases in screening, but the opportunity to adapt the brief intervention did not result in changes in the percentage of patients who were screened [43, 45]. When non-physician providers and physicians were exposed to the same implementation strategies, a higher percentage of patients were screened by non-physician providers than physicians [46].

##### Adoption

Two studies examined the adoption of screening by providers. One study found that physicians at the completion of the study were more likely to screen than at the beginning of the study. However, the adoption of screening was not significantly different between the intervention group and the control group in this study [47]. In contrast, another study found that providers who attended more training sessions were significantly more likely to screen patients for substance use than providers who attended fewer training sessions [48].

#### Brief intervention

##### Reach

Seven out of the 10 studies reporting outcomes related to the brief intervention measured the percentage of patients who received the brief intervention. Most of these studies (*n* = 5) used the same implementation strategies for all study participants using a quality improvement, pre-post, longitudinal study design, or retrospective design. The results of these studies differed; while the percentage of patients receiving the brief intervention significantly increased in one study [41], other studies demonstrated no change in reach [36, 38, 39], A retrospective study evaluating a new nationwide performance measure (supported by electronic decision support and financial incentives) demonstrated a significant increase in reach of the brief intervention. However, this study does not assess or describe implementation strategies used within each facility to promote the use of SBIRT [49].

The remaining studies on the reach of the brief intervention (*n* = 2) compared different implementation strategies between and among groups. In a randomized controlled trial, reach was higher in the intervention group than the control group, but it is not clear if this difference was statistically significant [44]. Adapting the intervention to allow for an electronic brief intervention did result in a significant increase in the percentage of patients who received a brief intervention overall [45].

##### Adoption

Three studies measured the adoption of brief intervention by providers. More providers reported using the brief intervention after exposure to the implementation strategies [47, 50], and providers who attended more training sessions were more likely to use the brief intervention than their peers who attended fewer training sessions [48].

#### Referral to treatment

##### Reach

Of the four studies reporting outcomes related to the percentage of patients who received a referral to treatment, most measured reach (*n* = 3). There was not a notable change in referral to treatment for two studies [39, 44], but Sterling et al. [48] found that embedding a behavioral health care practitioner into primary care resulted in a significantly lower percentage of patients receiving a referral to treatment than patients receiving usual care.

##### Adoption

In one study of provider adoption of referral to treatment, Salvalaggio et al. [47] noted a significant increase over time in the overall percentage of physicians reporting that they refer patients to treatment. This outcome, however, was not significantly different between the intervention and control groups.

#### Brief intervention/referral to treatment

##### Adoption

Mertens et al. [46] measured the outcome, brief intervention/referral to treatment, based on documentation of either a brief intervention or a referral to treatment. Evidence suggests that physicians may be more likely to provide a brief intervention/referral to treatment than non-physician providers, but the physicians in this study were also less likely to screen patients than non-physician providers [46].

#### SBIRT

##### Adoption

Two studies did not differentiate screening, brief intervention, and referral to treatment as three separate interventions, but instead asked providers about their use of SBIRT overall before and after exposure to implementation strategies. In both studies, providers reported an increase in the use of SBIRT [50, 51], although the reported use of SBIRT 12 months after the intervention was not as high as the reported use of SBIRT 3 months after the intervention [51].

## Discussion

SUD are common and detrimental to individuals and society as a whole. SBIRT, an approach to the prevention and treatment of unhealthy substance use, is not consistently implemented in healthcare settings. Different implementation strategies may be used to increase the delivery of SBIRT to patients or the use of SBIRT by providers, but there had not been a recent review of the evidence from multiple healthcare settings. This scoping review included 18 articles and was guided by the research question, “What implementation strategies are used to increase the reach and adoption of SBIRT when implementing SBIRT in healthcare settings, and what are the associated outcomes related to reach and adoption?”

The majority of the studies were conducted in the United States and focused on screening and providing a brief intervention for alcohol use in the emergency department and primary care settings. These study characteristics align with the recommendations for practice from the American College of Surgeons Committee on Trauma [52] and the U.S. Preventive Services Task Force [53]. There is a gap, however, in the existing literature about the implementation of SBIRT in acute care settings. When compared to the general population, patients admitted to the hospital have higher rates of SUD [1, 54]. McQueen and colleagues [55] contend that when a brief intervention for heavy alcohol is used in hospitalized patients, this intervention is associated with a reduction in alcohol consumption and death rates. Additionally, The Joint Commission quality measures for hospitalized adult patients support and recommend screening and providing a brief intervention for unhealthy alcohol use [56].

While most of the studies did not state a theory used to guide the study, each study described a multi-modal approach with the use of various strategies to support implementation. Numerous studies included strategies to train stakeholders and develop stakeholder interrelationships, but less attention has been given to adapting and tailoring SBIRT. There are core components of SBIRT that must remain the same to maintain fidelity to the intervention, but the peripheral components of SBIRT (e.g., who completes the screening, how the brief intervention is provided) can be adapted to fit the organizational context. Bendsten et al. [45] found that allowing providers to select between an electronic brief intervention or a face-to-face brief intervention was associated with an increase in the percentage of patients who received a brief intervention. SBIRT is a multi-step intervention that involves multiple professions and teamwork. More research about adapting the intervention or implementation process may be beneficial to increase the reach and adoption of SBIRT. Of note, only a few studies engaged patients or other consumers in the implementation process. Providers and patients report discomfort discussing substance use as a barrier to the implementation of SBIRT [57], but 95% of hospitalized patients reported that they would feel comfortable if a nurse discussed alcohol use with them [58]. There is a potential to enhance implementation by further researching adaptation of SBIRT and patient and consumer engagement.

When evaluating outcomes associated with the implementation of SBIRT, most of the studies evaluated organizational or group-level outcomes and did not evaluate provider-level outcomes. Nevertheless, the factors influencing individual providers’ decisions about the adoption of an intervention differ from the factors influencing organizational decisions [59]. Additionally, the use of SBIRT may increase initially and then decrease over time [42, 51], but there is a paucity of research on the sustainment of SBIRT. This review also revealed that the use of implementation strategies is generally associated with increases in the reach and adoption of screening, but evidence about the brief intervention is inconclusive, and evidence regarding the referral to treatment is scarce.

### Limitations

There are several limitations of this scoping review. Only one reviewer screened all of the titles and abstracts, and therefore some studies may have been inaccurately excluded from the study. The reviewers also extracted implementation strategies from each article and then selected categories for each strategy, but the categories selected by the reviewers may not reflect the actual intention of investigators in the original study. Furthermore, the authors of each article may not have described every implementation strategy used to support the implementation of SBIRT, and those strategies that were described may not have included all pertinent details. As the method did not include an appraisal of the quality of evidence, the results of this scoping review indicate gaps in the evidence but does not draw conclusions regarding the effectiveness of different implementation strategies.

## Conclusion

In summary, this scoping review provides a summary of the strategies used in healthcare settings to support the reach and adoption of SBIRT. Most of the empirical evidence about the implementation of SBIRT in healthcare settings is from studies conducted in the United States in primary care and emergency department settings. Additional research in other healthcare settings (such as acute care) may identify strategies to support the implementation of SBIRT in other contexts. Healthcare leaders and researchers often train and educate stakeholders and use strategies to develop stakeholder interrelationships, but there is a lack of empirical evidence about adapting the intervention or engaging consumers. Because implementation is more effective when strategies address patient, professional, and organizational factors, leaders should consider using a comprehensive approach that does not limit the focus to providers. Finally, researchers often measure the reach of screening and the brief intervention, with less focus on adoption of SBIRT by providers or reach and adoption of referral to treatment. Referral to treatment is a complex process, and strategies to implement screening and brief intervention within one healthcare interaction may differ from the strategies required to effectively refer a patient to treatment.

## Data Availability

Data sharing is not applicable to this article as no datasets were generated or analyzed during the current study.

## Abbreviations

SBIRT: Screening, Brief Intervention, Referral to Treatment
SUD: Substance use disorder
